# Targeting beliefs and behaviours in misophonia: A case series from a UK specialist psychology service

**DOI:** 10.1017/S1352465823000462

**Published:** 2023-10-19

**Authors:** Jane Gregory, Tom Graham, Brett Hayes

**Affiliations:** 1 Department of Experimental Psychology, University of Oxford; 2 Oxford Health NHS Foundation Trust; 3 South London and Maudsley NHS Foundation Trust; 4 Salomons Institute for Applied Psychology, Canterbury Christ Church University

**Keywords:** Case series, Imagery rescripting, Inhibitory learning, Mechanisms of change, Safety-Safetyseeking behaviours

## Abstract

**Background:**

Misophonia, a disorder of decreased sound tolerance, can cause significant distress and impairment. CBT may be helpful for improving symptoms of misophonia, but the key mechanisms of the disorder are not yet known.

**Aims:**

This case series aimed to evaluate individual, formulation-driven CBT for patients with misophonia in a UK psychology service.

**Methods:**

A service evaluation of one-to-one therapy for patients with misophonia (N=19) was conducted in a specialist psychology service. Patients completed an average of 13 hours of therapy with a focus on meaning applied to their reactions to sounds and associated behaviours. Primary outcome measures were the Misophonia Questionnaire (MQ) and the Amsterdam Misophonia Scale (A-MISO-S). Repeated measures t-tests were used to compare scores from pre-treatment to follow up, and reliable and clinically significant change on the MQ was calculated.

**Results:**

Scores significantly improved on both misophonia measures, with an average of 38% change on the MQ and 40% change on the A-MISO-S. From pre-treatment to follow up, 78% of patients showed reliable improvement on the MQ and 61% made clinically significant change.

**Limitations:**

Limitations included a lack of control group, small sample size, and the use of an outcome measure that had not been thoroughly validated for a treatment-seeking sample.

**Conclusions:**

These results suggest that one-to-one, formulation-driven CBT for misophonia is worth exploring further using experimental design. Potential mechanisms to explore further include feared consequences of escalating reactions, the role of safety seeking behaviours and the impact of early memories associated with reactions to sounds.

## Introduction

Misophonia is a disorder characterised by a decreased tolerance to certain sounds, typically repetitive, everyday sounds that most people would find neutral or mildly aversive (Jastreboff & Jastreboff, 2002; Swedo et al., 2022). Individuals with misophonia report significant distress and impairment, including problems with work, study and relationships (Edelstein et al., 2013; Rouw & Erfanian, 2018). Misophonia is also associated with increased symptoms of depression and anxiety (Rosenthal et al., 2022; Vitoratou et al., 2021).

There is currently no consensus on the best treatment approach to support individuals with misophonia (Swedo, et al., 2022). Developing and refining treatments requires understanding the key mechanisms contributing to the onset and maintenance of the disorder (Clark, 2004); these mechanisms have not yet been established for misophonia. However, there have been a range of cognitive and behavioural features reported by individuals with misophonia, suggesting that one of the lines of pursuit for treatment could be the development of a theoretical cognitive model which could then be systematically tested..

Cognitive features reported by individuals with misophonia include feeling like they might panic or explode (Singer, 2018; Vitoratou et al., 2021), lose control (Hocaoglu, 2018; Jager, de Koning, et al., 2020; Vitoratou et al., 2021) and not be able to cope (Schneider & Arch, 2017) if they are unable to get away from certain sounds. They report negative appraisals, including judgement towards the person making the sound (Edelstein et al., 2013; Reid et al., 2016; Vitoratou et al., 2021), a perception of oneself as being an unlikeable or angry person underneath for reacting to sounds (Vitoratou et al., 2021), and a feeling of failure and future hopelessness as a result of the condition (Edelstein et al., 2013; Rouw & Erfanian, 2018; Vitoratou et al., 2021).

Behaviours reported by individuals with misophonia include blocking sounds (Alekri & al Saif, 2019), avoidance (Alekri & al Saif, 2019; Johnson et al., 2013; Rouw & Erfanian, 2018; Schneider & Arch, 2017), post-event rumination (Muller et al., 2018) and verbal aggression (Hocaoglu, 2018; Reid et al., 2016). However, in the absence of experimental research or detailed case reports about the context, function and consequences of an individual’s behaviour, it is difficult to distinguish between adaptive coping strategies that improve symptoms and functioning and safety-seeking behaviours that maintain beliefs about threat or violation (Thwaites & Freeston, 2005).

Some of these features of misophonia have been targeted with cognitive behavioural therapy (CBT) and there is emerging evidence that CBT may be helpful for improving the symptoms and impact of misophonia. Case studies have described a range of CBT strategies used for misophonia, including challenging beliefs and assumptions (Bernstein et al., 2013; Muller et al., 2018), cognitive restructuring (McGuire et al., 2015; Reid et al., 2016; Singer, 2018), exposure-based exercises (Bernstein et al., 2013; McGuire et al., 2015; Muller et al., 2018; Reid et al., 2016; Singer, 2018) and attention training (Bernstein et al., 2013). Strategies geared towards non-judgmental acceptance of thoughts and feelings about sounds were also reported (Kamody & del Conte, 2017; Schneider & Arch, 2017).

An open trial of 90 patients found that misophonia symptoms improved following group CBT (Schröder et al., 2017). The interventions in this study included attention training, counterconditioning by pairing “trigger” sounds with positive pictures or videos, stimulus manipulation intended to gain a sense of control over sounds, and relaxation techniques. Participants were provided with seven or eight sessions of four hours in a group setting with a therapist and a co-therapist. Significant improvement was seen in 48% of participants, with improvement defined as a 30% or more reduction on the Amsterdam Misophonia Scale (A-MISO-S), a measure designed by the research team, and a final score on the Clinical Global Impression - Improvement Scale (CGI-I) of very much or much improved.

The open trial was followed by a randomized controlled trial of group CBT for misophonia (Jager, de Koning, et al., 2020). The content for this trial was similar to the open trial, with the addition of “re-evaluating (eating) norms and stress reduction”, and the treatment was offered over seven sessions of three hours plus a one and a half hour follow up. Reduction in scores on the Amsterdam Misophonia Scale-Revised (AMISOS-R) was significantly greater in the CBT group compared to the waitlist control, and 37% of the intention-to-treat sample reported very much or much improved on the CGI-I, compared to 0% in the waitlist control group.

The group CBT trials reported above used a modular approach, in that a set of modules were delivered to all participants. This kind of approach can useful for treating single disorders and in randomised trials to ensure consistency in the delivery of the intervention. However, it may be less helpful when treating patients with multiple disorders (Persons, et al., 2006) and inefficient for patients with disorders for which there is no empirical evidence for key mechanisms likely to be maintaining the problem (Clark, 2004). Considering the high co-morbidity of psychiatric disorders with misophonia (Rosenthal, et al., 2022) and the limited empirical evidence for key mechanisms maintaining misophonic distress, it may be more appropriate to target transdiagnostic mechanisms (Rosenthal et al., 2023).

One way of targeting the mechanisms specific to an individual patient is to use a case formulation-driven approach (also known as case conceptualization; Kuyken, et al., 2011), where the clinician and patient develop hypotheses about the mechanisms maintaining the individual’s problem and use evidenced-based strategies to target those mechanisms. This approach allows the clinician and patient to evaluate the therapy as they go and revise the formulation and treatment accordingly (Persons, 2005). Observations from formulation-driven clinical practice can then contribute to the development of a theoretical model for testing in experimental studies.

For newly characterised conditions such as misophonia, case series are an important step for demonstrating proof (or disproof) of concept and developing hypotheses to be tested in future research (Kempen, 2011). In a retrospective service evaluation, we aimed to assess whether symptoms of misophonia reduced following a course of formulation-driven CBT in a consecutive series of cases. We aimed to test whether there was reliable improvement and clinically significant change from pre-treatment to follow up. A secondary aim was to evaluate whether self-reported symptoms of anxiety and depression decreased following a course of misophonia-focused CBT in these individuals. Given the association between misophonia and symptoms of anxiety and depression, and the transdiagnostic approach of formulation-driven CBT, it was hypothesised that there would be a reduction in symptoms of misophonia, depression and anxiety.

## Method

This retrospective service evaluation included 19 patients from a National Health Service (NHS) specialist psychology service in the United Kingdom, for which misophonia was identified as the main problem contributing to their psychological distress. Referrals included those made directly to the service as well as referrals seen within the specialist service on behalf of local primary care psychology teams. Demographic information is summarised in Table 1.

Patients were included if they met the following criteria: a) identified as having clinically significant misophonia at assessment and this was the main issue of focus for therapy; b) at least 17 years old; c) not actively suicidal; and d) not receiving any concurrent psychological therapy. There were no exclusion criteria based on the presence of cooccurring conditions, but it was required that targeting misophonia was their focus for treatment.

Data for these 19 patients were collected over a two-and-a-half-year period, starting with the service’s first patient seen specifically for help with misophonia. In this timeframe, we received 22 referrals where misophonia was mentioned in the referral. For two of these referrals, misophonia was not identified as the main presenting problem and so these two patients were not offered treatment for misophonia after their initial assessment. One patient postponed treatment due to personal circumstances that meant they were unable to attend appointments at the clinic, for reasons unrelated to the treatment. All other patients who received any amount of therapy for misophonia (N=19) in this time frame were included in the evaluation. There were no other referrals for misophonia during the 2.5-year period.

These patients were seen as part of routine treatment provision and this case series was approved as a service evaluation by the *[Redacted for anonymity during review process]* Information Governance team.

### Measures

The primary outcome measures were the Misophonia Questionnaire (MQ; Wu et al., 2014) and the Amsterdam Misophonia Scale (A-MISO-S; Schröder et al., 2013), which were the two main published scales for misophonia at the time this group of patients were seen. The MQ (Wu et al., 2014) consists of three parts, the 7-item Misophonia Symptom Scale (Cronbach’s α = .86 in the author’s initial validation of the scale; Wu et al., 2014) and the 10-item Misophonia Emotions and Behaviours Scale (α = .86), both rated on a five-point scale from 0 (not at all true/never) to 4 (always true/always) and the single item Misophonia Severity Scale, rated from 1 (minimal) to 15 (very severe). The first two sections are combined to create the MQ total (α = .89), with a score ranging from 0 to 68. The A-MISO-S (Schröder et al., 2013) consists of 6 items, also rated from 0–4, with a total possible score of 24 (psychometric properties not available). Full psychometric analyses have not been published for either scale and the limitations of these measures have been described elsewhere (see, for example, Rosenthal et al., 2021).

Secondary outcome measures were the Patient Health Questionnaire-9 (PHQ-9; Kroenke et al., 2001), measuring depression symptoms using a 4-point scale (total score range of 0 to 27; α = .89; clinical cut-off >9), and the General Anxiety Disorder-7 (GAD-7; Spitzer et al., 2006) measuring anxiety symptoms, also on a 4-point scale (total score ranging from 0 to 21; α = .92; clinical cut-off >7).

### Procedure

#### Assessment

The diagnosis of misophonia and suitability for CBT was confirmed by either the first or second author for all patients, based on their initial assessment appointment. At the time we started seeing patients with misophonia, the only published diagnostic criteria available were those proposed by Schröder, et al. (2013), which included: the presence of an aversive reaction to specific sounds produced by a human being, which starts with irritation or disgust and progresses to anger, with a sense of loss of control; recognition by the person that the reaction is out of proportion to the stimulus; avoidance or enduring sounds with distress; causing significant distress or impairment, and not better explained by another disorder. We adapted these criteria based on our clinical observations and emerging research, including: that trigger sounds were not necessarily caused by other humans; that reactions did not necessarily start with irritation or disgust; that there was some experience of emotional, behavioural or physical dysregulation, but that anger did not need to be present; and that individuals may engage in safety-seeking behaviours other than avoidance to be able to encounter sounds. We also required that the symptoms had been problematic for at least one month, to allow for the possibility that temporary changes in responsivity to sounds could occur following changes in environment (e.g. construction sounds outside the home) or after an illness, injury or traumatic event. If symptoms started after such a change, but persisted for more than one month, then they would meet criteria for diagnosis. As is standard procedure for the service, all patients completed the primary and secondary outcome measures at the assessment, before each treatment appointment, and at follow up.

#### Intervention

All patients received one-to-one, formulation-driven cognitive behavioural therapy, aiming to reduce the distress and functional impact caused by misophonia. Potential mechanisms were identified from the individual’s formulation, and interventions were selected to target those theorised mechanisms. For most patients, the bulk of therapy was delivered as weekly one-hour sessions, but treatment delivery was also adapted to meet the needs of the patient, the therapy and the service. For example, some sessions involving processing of early memories were longer, some patients completed an intensive or semi-intensive format with fewer sessions of 2-4 hours each, and some had longer breaks between sessions to put things from therapy into practice or to meet other demands on their time. Variations in the total hours of treatment were the result of individual needs of the patient and the decision to end therapy was decided by the therapist and patient together. Information about the treatment format is summarised in Table 2.

All patients were seen by either the first or second author as their primary therapist, who both have professional doctorates in psychology and are accredited CBT therapists in the UK. Some sessions were observed by assistant psychologists or students on placement, who often participated in behavioural experiments. One patient had a second clinical psychologist join as co-therapist, which was done as part of that therapist’s training in treating patients with misophonia and has been written up separately as a case study *[reference redacted for blind review]*.

**Figure 1:**
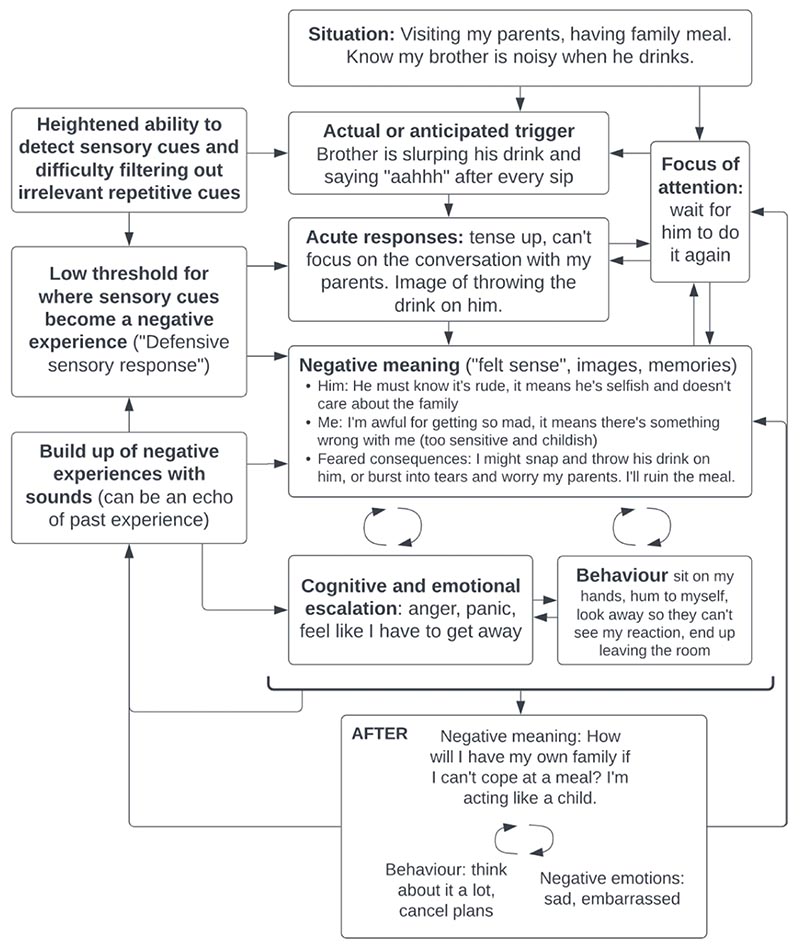
Example of an individualised case formulation of misophonia, based on themes emerging from the case series but not pertaining to a specific patient

In the first 2-3 hours of treatment, patients completed an individualised formulation of their difficulties. This was usually done by bringing to the surface a recent memory of being triggered by a sound, ideally one that felt typical of them and their misophonia. They were encouraged to tune into their bodies and the “felt sense” of what was happening (Butler et al., 2010; Gendlin, 1996), that is, what felt true to them in the moment, even if it did not appear as a verbal thought at the time. Our initial framework for mapping out the problem was Salkovskis’s “vicious flower”, drawing from its adaptation for transdiagnostic use for medically unexplained symptoms (Salkovskis et al., 2016). This model proposes that the intensity of reactions in the problem situation is influenced by misinterpretation of bodily sensations (or sensory information, in the case of misophonia), feared consequences and perceived capacity to cope. It poses that to treat the problem, we need to understand the maintenance processes causing those problem situations to persist over time. We identified some recurring themes during the case series that enabled us to adapt that model for misophonia (see Figure 1 for an example formulation, not based on any particular patient from the series).

Each patient developed competing explanations for their problem, using the “Theory A / Theory B” technique frequently used as part of formulation-driven CBT for obsessive compulsive disorder (OCD; Bream et al., 2017). This strategy provides alternative explanations for what keeps an individual’s problems going and identifies the related action that would be required for each possible explanation. These theories are then tested with a series of experiments and evidence is gathered to help the patient decide which theory is the best fit for their problem. From our patients’ individual formulations, it emerged that many people already had two competing theories, sometimes simultaneously feeling like their difficulties were caused by: A) a problem with other people; or B) a problem with me. We introduced a third explanation, that their difficulties were the result of C) a problem with sound sensitivity that had spiralled. Theory C included an understanding that there is wide natural variation in sensory responsivity, and that the distress and impairment associated with misophonia was influenced by a range of biological, experiential, systemic, emotional, behavioural and environmental factors.

Based on their individualised formulation, transdiagnostic CBT techniques were used to target the individual’s theorised maintenance cycles, including behavioural experiments, imagery rescripting, exposure-based experiments using a belief disconfirmation (Salkovskis et al., 2007) or expectancy violation (Craske et al., 2014) approach, attention training, behavioural activation, video feedback, interoceptive exposure, problem solving, and testing the value of coping strategies.

Behavioural experiments were used to identify, test and change the proposed maintenance cycles (Bennett–Levy et al., 2016). Experiments targeted both misophonia-specific beliefs (e.g. “If someone makes a sound they know I don’t like, they must not care about me”) and more general beliefs affecting their misophonia (e.g. “If I show my emotions, I will be rejected”). These could be beliefs that felt true in the moment, but didn’t necessarily feel true at other times (e.g. “They’re making that sound deliberately to hurt me”) and beliefs that were more stable across situations (e.g. “Having judgemental thoughts about loved ones makes you a bad person”). Behavioural experiments were also used to help distinguish adaptive coping strategies from safety seeking behaviours, by identifying and testing the function of behaviours, identifying unintended consequences, and testing out new behaviours for comparison. Table 3 shows some examples of the types of experiments used.

Imagery rescripting (ImRs) was used for processing early memories associated with their misophonia. This technique is increasingly being integrated into CBT and included in intervention studies for a range of disorders (Arntz, 2012). We used the protocol described by Arntz & Jacob (2012), using an affect bridge to connect the present experience to a memory (usually in childhood) associated with the current emotion, then rescripting the memory based on the previously unmet needs of the younger self. The function of ImRs is not to “rewrite” the memory, but to update the meaning associated with the memory. For misophonia, this included things like validating and comforting the child’s emotions, explaining the limitations of the other person present in the memory, or telling the child, “You are not crazy, you have something called misophonia”.

In the second half of therapy, patients completed experiments related to novel interaction with sounds and their reactions to sounds. These experiments involved some exposure to sounds, but not with the purpose of habituating to sounds. The patients decided when they were ready to try these experiments and each one was planned together in line with their formulation. The goals of experiments engaging with sounds were to create new associations with sounds (Craske et al., 2014; Frank & McKay, 2019), thus reducing the intensity of the “felt sense” feeling of threat and violation, to test feared consequences and beliefs about capacity to cope and to test out and compare coping strategies. The experiments typically involved short exposure to sounds at a time (20-30 seconds), followed by rating the sense of threat of violation, then repeating the experiment several times. The experiment was terminated if it caused more distress than “real life” exposure to sounds, or if there was no change after 2-3 repetitions.

Sound-based experiments included moving closer to sounds to test predicted outcomes, engaging with sounds by labelling the acoustic features of the sounds, “inviting” more sounds to shift beliefs about violation and control, and playing with sounds electronically or by using a pretend remote control on the person making sounds. We used humour and imagery to shift the perception of threat and violation, adapting strategies described by Frank and McKay (2019). For example, one person imagined wind-up chattering teeth toys during meals, another pictured rain on a roof while listening to keyboard sounds, another put on a brief comedy sketch in session, portraying what they imagined the person making a recurring sound must be doing to be able to create such a noise. Some patients conducted orchestras of mouth sounds and held competitions for who could make the loudest sounds. Table 3 includes some other examples of experiments involving sounds to test a particular theory or compare coping strategies.

A range of other strategies were used depending on the individual formulation. For example, one person identified that job dissatisfaction had coincided with worsening misophonia symptoms, for which we used problem solving strategies. We used behavioural activation, attention training and grounding techniques for individuals who were ruminating on the problem or took a long time to recover after a trigger event. For those struggling with anger about circumstances outside of their control, we used techniques weighing up the pros and cons of holding onto anger and writing anger letters. Skills in communication, assertiveness, tolerance and setting boundaries were provided for patients whose misophonia was connected to challenges in relationships.

Finally, patients wrote up a therapy blueprint. This included a description of changes made in therapy, ideas for maintaining progress, and a plan for dealing with distress and setbacks. This plan also included an understanding of their sensory sensitivity and the healthy coping strategies and accommodations that they would continue to use, when required for focus, relaxation and enjoyment of activities.

### Statistical analyses

Repeated measures t-tests were used to compare mean scores at pre-treatment and follow up on primary and secondary measures. “Pre” scores refer the first time they completed the outcome measures for our service, which may have been before or after their assessment, and always prior to commencing active treatment.

To reduce the number of comparisons tested, we did not calculate differences between start and end of treatment, only between pre-treatment and follow up. We made a Bonferroni adjustment to account for multiple comparisons, with statistical significance determined at *p*<.008. Where there was missing data on any measure for either the pretreatment or the follow up, patient data was not included in any of the calculations or comparisons for that particular measure, but was retained in calculations for the other measures. For example, if a patient had missing follow up data for the GAD-7 but complete data for other measures, their scores were not included in calculating means for GAD-7 at pre-treatment nor follow up, but they were included in all other analyses.

The Leeds Reliable Change Indicator (Morley & Dowzer, 2014) was used to calculate reliable change and clinically significant change and to graph reliable change.. The MQ was the best validated misophonia scale available, for which clinical and comparison norms had been published (Wu et al., 2014), at the time of collecting data. Therefore, we selected this measure to calculate the number of patients who showed reliable change and clinically significant change (Jacobson & Truax, 1991) from baseline to follow up. We selected criterion b for clinically significant change, which is one of the two options recommended when both clinical and comparison norms are available (Jacobson & Truax, 1991). This criterion states that change is clinically significant when the individual has showed reliable change and their final scores fall within the range of the “functional” population (i.e. within two standard deviations of the mean of the comparison group). We chose this over criterion c, which is when a patient moves to closer to the mean of the comparison group than to the mean of the clinical group by the end of therapy. The rationale for this choice was that the mean score for our patients (i.e. treatment-seeking) at baseline was over 30% higher than the mean for the “clinical” group for the study in which clinical and comparison norms were reported (from participants in a university sample scoring above cut-off on a single item rating severity, which has not been psychometrically evaluated). This difference in means suggested that our patient group experienced more severe symptoms compared to the group for which “clinical” norms were available. We decided that our patients’ scores at the end of treatment in relation to the comparison group norms would therefore be a better criterion for clinically significant change.

## Results

There was missing data for one patient on the MQ at pre-treatment, and for three patients on the GAD-7 and PHQ-9 at follow up. Therefore, their data were not included on any analyses using those specific measures but were included in analyses for other measures.

### Comparisons on misophonia measures

For both misophonia measures, t-tests revealed a significant difference on score from pre-treatment to final follow up (Table 4) with large effect sizes (Cohen’s *d* >.08). There was a 38% change on the MQ total score. For the two subscales of the MQ, there 21% change on the Symptoms Scale and 51% change on the Emotions and Behaviour Scale. There was a 40% change on the A-MISO-S.

### Reliable and clinically significant change on the Misophonia Questionnaire (MQ)

**Figure 2:**
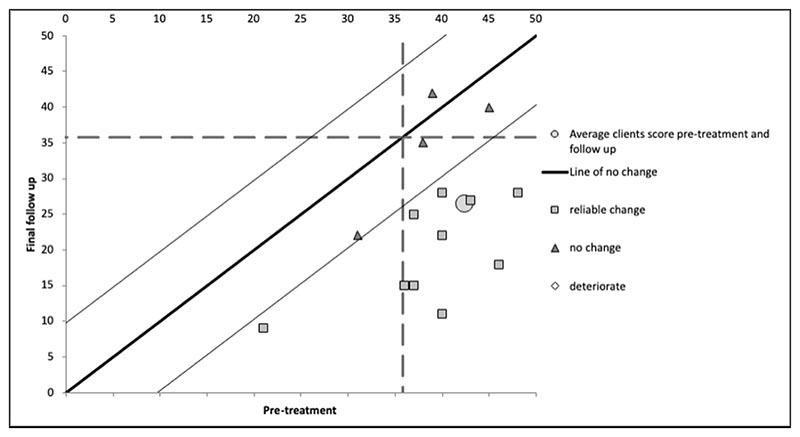
Graph of reliable change index for Misophonia Questionnaire from pre-treatment to follow up

From pre-treatment to follow up, 14 out of 18 patients (78%) had reliably improved on the MQ, and 4 had not made reliable change. There was no reliable deterioration found. Eleven patients (61%) made clinically significant change. The average score on the MQ before treatment was 42.39 (SD = 9.39) and the average score at follow up was 26.44 (SD = 11.18). Patient scores on the MQ can be seen in Figure 2, with pre-treatment scores on the x-axis and follow up scores on the y-axis. Patient markers within the tramlines indicate no reliable change, and those below and to the right of the tramlines indicate reliable change.

### Comparisons on depression and anxiety measures

Mean scores for symptoms of depression and anxiety were significantly reduced from pre-treatment to follow up (Table 5), with large effect sizes (Cohen’s *d* >.08).

Mean scores for depression symptoms on the PHQ-9 moved from the mild symptom range (score range 5-9; Kroenke et al., 2001) to non-clinical (<5). For anxiety symptoms, mean GAD-7 scores reduced from the clinical range to the non-clinical range (with a clinical cut-off score of >7; Spitzer et al., 2006).

## Discussion

This case series aimed at evaluating whether one-to-one, formulation-driven CBT significantly reduced symptoms of misophonia in patients at a specialist psychology service. Close to 80% of patients showed reliable improvement and more than half showed clinically significant change in misophonia symptoms. Symptoms of depression and anxiety also significantly reduced.

This study adds to the emerging literature on psychological treatments for improving symptoms of misophonia, which has consisted mostly of descriptive case studies of individual CBT (Bernstein et al., 2013; McGuire et al., 2015; Muller et al., 2018) and evaluations of group CBT in an open trial (Schröder et al., 2017) and a randomised controlled trial of group treatment (Jager, Vulink, et al., 2020). While misophonia is not classified as a psychiatric disorder (Swedo et al., 2022), it is clear that there are psychological aspects to distress in misophonia, and that CBT shows promise as an intervention.

Comparisons between our results and previous trials must be made cautiously due to the differences in aims, methodology, measurement tools and criteria used for clinical improvement. We noted that from pre-treatment to follow up, our group had a mean score change on the A-MISO-S of –6.1 points, with an average symptom improvement of 40%. In the open trial of group CBT, Schröder, et al. (2017), results showed a mean score change of –4.5 points pre- to post-treatment, with an average symptom change of 33%. It was interesting to note that in both our evaluation and the open trial, the average final score on the A-MISO-S was 9.1. Schröder, et al. (2013) found that higher initial scores on the A-MISO-S was predictive of greater symptom change, so it’s possible that our slightly increased symptom change was influenced by the higher average score at baseline (15.1 in our evaluation compared to 13.6 in the open trial). One important difference between our case series and the open trial was the total number of hours of treatment. Our patients had an average of 13 hours of treatment, compared to 28–32 hours of group treatment in the open trial. Considering we do not yet understand the key mechanisms of misophonia, it makes sense that individual therapy would be more efficient in terms of patient time, as the formulation-driven approach allows for targeting the maintenance cycles identified for that individual, without needing to spend time on interventions that may apply to other group members.

The relative change in scores between the two subscales of the MQ was an interesting finding. Most of the change in MQ scores can be accounted for by change on the Misophonia Emotions and Beliefs Scale, which measures how respondents react to sounds. There was very little change on the Misophonia Symptom Scale, which asks respondents to rate how sensitive they are to certain sounds, compared to other people (Wu et al., 2014). During informal feedback, some of our patients told us that even when they felt that their misophonia had improved substantially, and that their reactions were less intense, they still considered themselves to be sensitive to sounds compared to other people. This highlighted the limitations of this particular tool to measure change in change in misophonia.

Subsequent to this study, the S-Five, a valid and reliable tool for measuring misophonia, has been published (Vitoratou et al., 2021). The five dimensions of the S-Five capture aspects of misophonic severity that one might expect to shift with therapy, such as appraisals, aggressive outbursts, dysregulation in response to sounds and perceived impact (Vitoratou et al., 2021). We now use the S-Five to monitor outcomes, selected over the Duke Misophonia Questionnaire (DMQ; Rosenthal, et al., 2021) because of the five dimensions of the S-Five, which include cognitive appraisals that might be targets for CBT (e.g. beliefs that there is something wrong with oneself because of one’s reactions to sounds, and beliefs that the source of one’s distress is the bad behaviour of others). The DMQ includes a “cognitive response subscale”, but the items contained within the subscale seem to reflect a verbalisation of distress or dysregulation (e.g. “I am helpless” and “I would do anything to make it stop”) rather than an appraisal of the problem, and so may be less helpful for detecting different aspects of change during therapy. One patient from the present case series, whose treatment commenced after the S-Five became available, completed the S-Five at each session, and there were different points of change on the different factors for that particular patient, with scores on “internalising appraisals” (attributing blame to oneself) changing after the initial assessment, and “emotional threat” (a sense of, or prediction of, emotional dysregulation) scores changing after active treatment commenced *[Reference redacted for blind review]*.

Misophonia-specific interventions were those that included interacting with sounds and reactions in novel ways without using their usual coping strategies, to create new associations with sounds and test feared consequences. For some patients this involved addressing a specific, verbalised belief (e.g. “If I have to keep listening to this disgusting noise, I will throw up), for others it was simplified by rating their felt sense of threat and/or violation in the moment. The experiments were typically brief and repeated several times, with changes observed in the ratings of threat/violation between repetitions. Several patients gave positive feedback on the value of these experiments, saying their reactions felt fundamentally different afterwards (e.g. from intense violation to mild irritation), which could indicate that inhibitory learning had taken place (Craske et al., 2014). Further research is needed to determine the mechanism of change happening in these experiments.

Imagery rescripting (ImRs) was used to process and “update” early memories associated with the development of strong reactions to sounds. While trauma-informed, this technique can also target non-trauma memories that are meaningful in terms of the development of beliefs underlying the current problem (Holmes et al., 2007). While ImRs has not previously been reported in the context of misophonia, there is evidence that eye movement desensitization and reprocessing (EMDR) therapy, which involves a similar strategy of updating memories, may help reduce symptoms of misophonia (Jager et al., 2021). While the mechanisms behind ImRs are not fully understood, Kunze et al. (2019) proposed that there may be multiple mechanisms involved, and that there are likely different mechanisms are involved for ImRs techniques compared with exposure techniques.

From our clinical observations and informal feedback from patients, we noticed that there was variation in the change that resulted from ImRs. For some, it shifted the feeling that there was something seriously wrong with them for reacting that way (Theory B). For others, it helped to update beliefs from believing they don’t care (Theory A) to believing they don’t understand. For some, it was useful for distinguishing early experiences from current experiences (e.g. being able to leave the situation as an adult but not as a child; no longer being in a chaotic or abusive environment; improved skills in emotion regulation and impulse control as an adult), which strengthened their belief that distress in misophonia was compounded by their circumstances and therefore could potentially change (Theory C). Techniques to identify sensory triggers and distinguish between current (benign) circumstances and past traumatic circumstances (stimulus discrimination; Ehlers et al., 2004) were also used to support this differentiation at a felt-sense level. It would be useful to explore the relevance of early memories in misophonia using in-depth qualitative studies and to experimentally test ImRs as an intervention.

One of our aims was to develop hypotheses about potential cognitive and behavioural mechanisms that may be key to misophonia. We noticed some themes in terms of cognitions: beliefs that the individual is flawed or damaged in some way because of the way they react to sounds; beliefs that others who make problematic sounds are uncaring, thoughtless or willingly causing harm; and fears about what might happen in the moment (e.g. losing control or being judged) or longer term (emotional, physical or social harm caused by misophonia). We theorised that behaviours were intended to minimise emotional or behavioural dysregulation, prevent specific feared consequences (e.g. hiding emotions to avoid being judged), or to communicate distress. While we were able to work with individuals to test whether coping strategies achieved their intended function and to examine unintended consequences, no clear themes emerged across the group about whether particular kinds of behaviours might play a key role in maintaining distress.

Further research is needed to understand the role of behaviour in the development and maintenance of misophonia. Avoidance behaviour is a key maintenance factor in many anxiety disorders and treatment frequently aims to reduce avoidance (Butler, et al., 2010). To date, there have been no experimental or prospective studies examining whether avoidance is associated with worsening misophonia symptoms. One study (Dibb and Golding, 2022) found that higher avoidance of triggers was cross-sectionally associated with poorer quality of life in one domain (physical functioning) but, at 6-month follow up, higher avoidance of triggers at baseline predicted *higher* quality of life in another domain (role limitations as a result of emotional problems). This suggests that it is possible that some avoidance behaviour may actually help reduce the impairment associated with misophonia. Future studies need to assess a range of misophonic behaviours and explore the impact on short- and long-term misophonia severity and impairment.

## Strengths and limitations

One of the strengths of this case series is that it shows outcomes from highly individualised treatment, reflecting the “real world” therapy available to these patients. It used a consecutive referral design, showing the results of all eligible patients who underwent treatment in the relevant time period, including those who did not improve. There were no dropouts in the case series, other than one patient who postponed treatment for practical reasons, indicating a high level of acceptability for this treatment.

This was an uncontrolled case series evaluating the treatment provided in a healthcare service, rather than an experimentally designed piece of research, and therefore there are several limitations to be acknowledged. Firstly, our small sample size was predominantly female and white British, and there was no control group. Treatment was highly individualised and therefore there was no set “protocol” being evaluated here. The treatment format and number of sessions were not consistent across patients. The follow up period was not consistent, and some patients were seen for follow up sessions, while the follow up for others consisted only of submitting outcome measures. The results should therefore be considered exploratory and hypothesis-generating for future research.

At the time these patients were seen, the measures that were publicly available had not been subjected to rigorous psychometric evaluation and norms for a treatment-seeking population were not available for the best-validated measure. This limited our analysis to using non-clinical comparison groups for the assessment of clinically significant change. Future treatment studies should use the measurement tools for misophonia now available that are multidimensional and have been subjected to rigorous psychometric evaluation (e.g. Rosenthal et al., 2021; Vitoratou et al., 2021; Rinaldi et al., 2022), with both clinical (Vitoratou et al., 2021) and comparison (Vitoratou et al., 2023) norms available. Finally, while there was a significant decrease in scores for symptoms of depression and anxiety, it should be noted that at the beginning of treatment the average scores for both were in the mild range. Therefore, any hypotheses about the impact of misophonia-focused treatment on symptoms of depression and anxiety should be held lightly and tested further.

## Conclusions

Case series are an important early step in the development of research hypotheses and “proof of concept” for treatment of newly characterised conditions (Kempen, 2011). The present study demonstrates that further investigation of individual CBT as a treatment for misophonia is warranted. It also provides clinical observations that can contribute to the development of a theoretical model, which could then be tested in a series of experimental studies (Clark, 2004).

## Figures and Tables

**Table 1:** Summary of patient demographic information

Variable	Frequency	Percent
**Gender**		
Female	17	89.47
Male	2	10.53
**Ethnicity**		
Asian British or mixed white and Asian (Indian)	2	10.60
British - mixed unspecified	1	5.26
Black British or mixed white and Black Caribbean	2	10.60
White British	14	73.68

	Min	Max	Median	Mean	SD
**Age**	17.6	51.0	30.5	33.57	9.32

**Table 2:** Summary of information about treatment format

Variable	Min	Max	Median	Mean	SD
**Treatment details**					
Total therapy hours	8	19	12	13.00	2.75
Hours of treatment	8	18	12	12.42	2.76
Hours of follow up	0	2	0	0.58	0.77
Total number of sessions	5	19	12	12.37	3.18
Treatment sessions	5	18	12	11.74	3.05
Follow up sessions	0	2	0	0.63	0.76
Follow up period (weeks)	5	20	9	10.06	4.40

	Frequency	Percent
**Treatment format**		
In person only	11	57.89
Mixed online and in person	3	15.79
Online only	5	26.32
**Number of follow up sessions**		
Zero	11	57.9
One	5	26.3
Two	3	15.8

**Table 3:** Examples of behavioural experiments for misophonia

Theory being tested	Behavioural experiment
Having judgemental thoughts about others means I am a bad person	Survey to find out what proportion of people have judgemental thoughts or opinions about others
I can’t cope with strong emotions	Watching emotionally-charged videos (positive and negative emotions) to find out what happens when experiencing strong emotions
Making that sound means you are uncouth and don’t care about others around you	Discussion with others to explore possible reasons for making certain sounds, and considering consequences that some people may have if they don’t make those sounds
I might act on my violent imagery if I don’t do something to stop them	Deliberately bringing on violent images and test whether that leads to violent action
If I get too angry, my heart will beat too fast and might explode	Running up and down stairs to induce increased heart rate to test predictions
I will be judged if I tell people about how angry some sounds make me	Testing the waters by talking with friends or family members to see how they react
I look repulsed when I hear sounds and so I need to try to suppress my emotions	Using video feedback to find out if one’s reaction looked the same from the outside as imagined, then compare with how it looks when trying to suppress
Blocking my ears is the best way to reduce my anger	Comparing blocking ears with labelling the physical properties of the sounds
Glaring or scowling is the best way to get someone to stop making a sound	Monitoring the impact of glaring (effectiveness in stopping sounds and unintended consequences)
I should try and push away my feelings or they will be overwhelming and obvious	Comparing emotion suppression with emotion labelling
I must escape the sounds of my feelings will escalate beyond my control	Trying “opposite action”: touching the wall to feel the vibrations of neighbour’s sounds; smiling at a person who is making a trigger sound; invite the person to make the sound more and louder (either imagined or out loud with a willing participant)

**Table 4:** Comparisons between pre-treatment and follow up measures of misophonia symptoms

Measures	Pre-treatment		Final follow up		t	df	p (two-sided)	Cohen’s *d*
	Mean	SD	Mean	SD				
Misophonia Questionnaire	42.39	9.39	26.44	11.18	6.90	17	<.001	1.63
MSYS	18.44	5.08	14.61	5.56	3.60	17	.002	0.85
MEBS	23.94	5.20	11.83	6.30	7.95	17	<.001	1.87
A-MISO-S	15.11	2.89	9.05	3.24	6.78	18	<.001	1.55

**Note**. MSYS = Misophonia Symptom Scale (MQ subscale 1) MEBS = Misophonia Emotions and Beliefs Scale (MQ subscale 2) A-MISO-S = Amsterdam Misophonia Scale A Bonferroni adjustment was applied for multiple comparisons and all results were significant at *p*<.008

**Table 5:** Comparisons between pre-treatment and follow up measures of anxiety and depression

Measures	Pre-treatment		Final follow up		t	df	p (two-sided)	Cohen’s *d*
	Mean	SD	Mean	SD				
Depression (PHQ-9)	8.50	*4.31*	4.50	*2.50*	3.40	15	.004	0.85
Anxiety (GAD-7)	9.63	*4.32*	5.63	*3.10*	4.59	15	<.001	1.15

**Note**. PHQ-9 = Patient Health Questionnaire-9, measuring symptoms of depression in the past two weeks GAD-7 = Generalised Anxiety Questionnaire-7, measuring symptoms of anxiety in the past two weeks A Bonferroni adjustment was applied for multiple comparisons and both results were significant at *p*<.008
